# Influence of patient symptoms and physical findings on general practitioners' treatment of respiratory tract infections: a direct observation study

**DOI:** 10.1186/1471-2296-6-6

**Published:** 2005-02-07

**Authors:** Thomas Fischer, Susanne Fischer, Michael M Kochen, Eva Hummers-Pradier

**Affiliations:** 1 Department of General Practice, Georg-August-University, Goettingen, Germany

## Abstract

**Background:**

The high rate of antibiotic prescriptions general practitioners (GPs) make for respiratory tract infections (RTI) are often explained by non-medical reasons e.g. an effort to meet patient expectations. Additionally, it is known that GPs to some extent believe in the necessity of antibiotic treatment in patients with assumed bacterial infections and therefore attempt to distinguish between viral and bacterial infections by history taking and physical examination. The influence of patient complaints and physical examination findings on GPs' prescribing behaviour was mostly investigated by indirect methods such as questionnaires.

**Methods:**

Direct, structured observation during a winter "cough an cold period" in 30 (single handed) general practices. All 273 patients with symptoms of RTI (age above 14, median 37 years, 51% female) were included.

**Results:**

The most frequent diagnoses were 'uncomplicated upper RTI/common cold' (43%) followed by 'bronchitis' (26%). On average, 1.8 (95%-confidence interval (CI): 1.7–2.0) medicines per patient were prescribed (cough-and-cold preparations in 88% of the patients, antibiotics in 49%). Medical predictors of antibiotic prescribing were pathological findings in physical examination such as coated tonsils (odds ratio (OR) 15.4, 95%-CI: 3.6–66.2) and unspecific symptoms like fatigue (OR 3.1, 95%-CI 1.4–6.7), fever (OR 2.2, 95%-CI: 1.1–4.5) and yellow sputum (OR 2.1, 95%-CI: 1.1–4.1). Analysed predictors explained 70% of the variance of antibiotic prescribing (R^2 ^= 0,696). Efforts to reduce antibiotic prescribing, e.g. recommendations for self-medication, counselling on home remedies or delayed antibiotic prescribing were rare.

**Conclusions:**

Patient complaints and pathological results in physical examination were strong predictors of antibiotic prescribing. Efforts to reduce antibiotic prescribing should account for GPs' beliefs in those (non evidence based) predictors. The method of direct observation was shown to be accepted both by patients and GPs and offered detailed insights into the GP-patient-interaction.

## Background

Over-prescribing in respiratory tract infections (RTI) has been the topic of numerous studies in general practice. Explanations for the overuse of antibiotics despite weak scientific evidence are multifaceted and vary from efforts to protect patients from "complicated" courses of disease to assumed patient expectations and to inadequate knowledge of physicians [1-4]. Recent investigations demonstrated that patient symptoms and physical findings were also associated with antibiotic prescribing [5,6]. A qualitative study using focus groups reported that general practitioners (GPs) tried to distinguish between viral and bacterial infection by history taking and physical examination [7]. This is in line with a questionnaire-based self-registration study showing a positive correlation between antibiotic prescribing and diagnoses/symptoms assumed to be associated with bacterial infection (e.g. 'sinusitis', 'tonsillitis' or 'yellow-green sputum') [8]. The antibiotic prescription rate in this study, however, was rather low (28%) and only few predictive items could be detected. Because self-registration studies are methodically limited (varying documentation quality, predetermining questionnaire items and completion of the forms in the sense of "scientific acceptability" [9], we designed a direct observation study to gain detailed insights into the influence of patient symptoms and physical examination on GPs' therapeutic decision. Additionally, we expected to obtain information about the doctor-patient-dialogue (e.g. about recommended self-medication or behaviour advice).

## Methods

We performed a structured, direct observation-based study. This concept is borrowed from social and cultural anthropology developed in the early 1960s [10]. Starting from an unstructured (qualitative) approach, which enabled open, unbiased data collection, a more systematic, structured concept using checklists (quantitative approach) was developed over time, particularly in nursing research [11-13]. We developed a checklist to record information on the interaction between GPs and patients with suspected RTI. Items included were based on history taking and physical examination protocols and the checklist was evaluated and adapted in a pilot study.

Data collection focused on patient complaints, results of physical examinations, further diagnostic procedures and diagnoses. Physical examination findings were coded according to the level of precision obtained (from 'pathological finding' to 'coated tonsils'). The registration of medication included prescriptions as well as recommendations for over-the-counter medicines (OTC) and the dispensing of drug samples (previously brought in by pharmaceutical representatives). Data were mostly acquired through observation during the consultation.

Potentially participating GPs were randomly selected from a GP register and addressed with a form letter. Of 62 GPs addressed, 30 participated this study (16 located in a medium-sized town in Lower-Saxony and 14 in rural areas of North Rhine-Westphalia). To avoid biasing GPs' behaviour no specific hypotheses were shared with the participants.

GPs were visited for one day by S. F. (medical student at time of observation) during a "cough and cold" period in winter; and all consecutive patients (age above 14 years) with symptoms consistent with RTI were included. All patients were informed that a medical student wanted to participate in the consultation and all of them accepted her attendance.

SAS software (Version 8.1) was used to analyse the data [14]. Respiratory tract infections were classified according to the International Classification for Primary Care (ICPC) [15]: 'upper respiratory tract infection (URTI)/common cold' (R74), 'sinusitis' (R75), 'tonsillitis' (R76), 'laryngitis' (R77) and 'bronchitis' (R78). Since exacerbations of chronic lung diseases are not defined in ICPC, we constructed a dummy variable (including R91, R95 and R96). Multiple diagnoses were accepted and all diagnoses were recorded as stated after the consultation by the GPs. Multiple logistic regression models were used to test for associations between patient characteristics, symptoms, diagnoses and recommended treatment (stepwise backward elimination, α = 0,05). Degree of effect is reported as odds ratios (OR) with 95% confidence intervals (CI). The most frequent patient complaints (cough, sneezing, sore throat, headache, fever, fatigue, hoarseness, myalgia, earache and facial pain) and pathological findings in physical examination as well as age, smoking status and duration of symptoms were included as predictive variables in the multivariate analyses. Drugs were classified using the Anatomic-Therapeutic-Chemical Classification (ATC) codes [16].

## Results

### Doctors and patients

GPs' median age was 48 years, their median experience in general practice was 12 years and 17% of the GPs were female. A total of 273 patients (51% women) were included (representing 21.4% of all patients visiting their GP at the time of observation). The median number of included patients per GP was 9. No patient refused participation. Patients' median age was 37 years (range 14 to 88).

### Diagnoses

Upper respiratory tract infections/common cold (URTI), bronchitis, tonsillitis and sinusitis were the dominating diagnoses and 88% of all patients got at least one of these diagnoses( Table 1). In total 319 diagnoses were made (40 patients were assigned 2 diagnoses and 3 patients 3 diagnoses).

**Table 1:** Frequency of diagnoses

**Diagnoses**	**No. of patients**	**(% of 273 patients)**
URTI/common cold	117	(42.9)
Bronchitis	70	(25.6)
Sinusitis	33	(12.1)
Tonsillitis	30	(11.0)
Acute exacerbation chronic lung diseases	24	(8.8)
Otitis media	17	(6.3)
Laryngitis	12	(4.4)
Other	16	(5.9)

(multiple diagnoses possible, URTI = upper respiratory tract infection)

### Self-medication and non-medical therapy

49 patients (18%) were asked by their GPs about self-medication and 83% of them acknowledged the previous use of OTC- drugs (predominantly symptomatic cough and cold drugs, particularly mucolytics). In 40% of these patients, GPs prescribed an OTC-preparation of the same ATC-classification. The use of household remedies was addressed in 12% of the patient encounters and three-quarter of the patients confirmed that they tried them before the consultation (inhalation in 36%, gargling in 27% and drinking of tea in 15%).

### Drug treatment

On average 1.8 (95%-CI: 1.68–1.95) drugs were prescribed per patient (Table 2). Only 12% of the patients left the consultation without a drug prescription, 27% received prescriptions for 3 and more drugs. The most frequently used drugs were acetylcysteine (89 prescriptions) and ambroxole (43 prescriptions). Only 8 patients asked for a specific prescription by themselves and 4 of them received the favoured drug. In total, 17 pharmaceutical samples were handed out to 14 patients (mostly cough and cold preparations). The recommendation to buy an OTC-preparation was given to 7 patients (mostly paracetamol).

**Table 2:** Drug Treatment (according to ATC-code)

**Main groups**	**Patients (%)**
Cough and cold preparations (R05)	208 (76.2)
Expectorants, excl. combinations with cough suppressants (R05C)	163 (59.7)
Other cold combination preparations (R05X)	57 (20.9)
Cough suppressants, excl. combinations with expectorants (R05D)	41 (15.0)
Cough suppressants and expectorants, combinations (R05F)	14 (5.1)
Antibacterials for systemic use (J01)	134 (49.1)
Macrolides and lincosamides (J01F)	52 (19.0)
Beta-lactam antibacterials, penicillins (J01C)	27 (9.9)
Cough suppressants and expectorants, combinations with antibacterials^(*) ^(R05G)	25 (9.2)
Other Beta-lactam antibacterials (J01D)	19 (7.0)
Tetracyclines (J01A)	7 (2.9)
Quinolone antibacterials (J01M)	4 (1.5)
Nasal preparations (R01)	43 (15.8)
Anti-Asthmatics (R03)	21 (7.7)
Throat preparations (R02)	10 (3.7)
Other (Breast unctions (R04), Echinacea-preparations (L03), otologicals (S02))	18 (6.6)

(^(*)^mostly tetracyclines, ATC = Anatomic, Therapeutic, Chemical Classification)

### Predictors of prescriptions

As shown in table 3 an association between specific diagnoses and antibiotic prescription rates could be demonstrated. Antibiotic prescription rate was highest in patients with the diagnosis 'tonsillitis' (90%) and relatively low in patients with the diagnosis 'common cold' (18%). The prescription rate of cough and cold preparations was above 90% in all diagnoses except 'tonsillitis'.

**Table 3:** Prescription rates of antibiotics and cough and cold preparations

**Diagnoses**	**Prescription rates (in %) of**	
	**Antibiotics [95% CI]**	**Cough and cold preparations [95%CI]**
Tonsillitis	89.7 [78.6–99.3]	55.2 [37.1–73.3]
Bronchitis	77.1 [67.3–87.0]	94.3 [85.3–95.9]
Laryngitis	75.0 [50.5–99.5]	91.7 [76.0–100.0]
Sinusitis	63.6 [47.2–80.0]	90.9 [81.1–100.0]
Acute exacerbation chronic lung disease*	50.0 [30.0–70.0]	95.8 [87.8–100.0]
URTI/ common cold	18.0 [11.0–24.9]	90.6 [85.3–95.9]

all patients	48.5 [42.6–54.5]	87.9 [84.0–91.7]

(URTI = upper respiratory tract infection, *see methods)

Multiple logistic regression analyses demonstrated associations between patient complaints, physical examination results and the prescription of *antibiotics *(Table 4). The calculated model explained 70% of the variance of antibiotic prescribing (R^2 ^= 0,695). Patients' age, smoking status and symptoms such as cough, sore throat, hoarseness, sputum, sneezing, headache and earache had no influence. Also, GPs' characteristics influenced the prescription rate: Although patients' age was uniformly distributed between younger and older GPs, younger GPs (<50 years) prescribed fewer antibiotics than older ones did (37.3 versus 54.4% of the patients, p < 0.05). Neither the number of treated patients (measured as number of individual patients treated per quarter of a year) nor the GPs' experience in general practice (measured as time of practicing) had influence on antibiotic prescription rates.

**Table 4:** Influence of patient complaints and physical examination results on antibiotic prescription

**Complaints/Physical examination results**	**OR**	**95%-CI**
Pathologically altered tonsils in mouth and throat inspection	15.41	3.6–66.16
Pathological otoscopy findings	8.85	1.16–67.58
Pathological cervical lymph node palpation findings	6.24	1.97–19.71
Rales in lung auscultation	4.29	2.09–8.83
Pathological results in paranasal sinus palpation (sinus tenderness)	3.20	1.38–7.42
Fatigue	3.09	1.42–6.72
Wheezing in lung auscultation	2.91	1.17–7.23
Fever	2.19	1.06–4.54
Yellow sputum	2.10	1.07–4.14

(multiple logistic regression, OR = Odds ratio, CI = Confidence interval, for included variables see methods)

*Cough and cold preparations *were associated with symptoms such as cough (OR 5.09, 95%-CI: 2.43–10.69), headache (OR 2.43, 95%-CI: 1.02–5.83), sneezing (OR 2.39, 95%-CI: 1.2–4.76), and abnormal findings in throat examination (OR 2.16, 95%-CI: 1.11–4.2). The calculated model explained 19% of the variance of cough and cold preparation prescriptions (R^2 ^= 0.186).

### Non-pharmaceutical treatment

Recommendations for non-pharmaceutical treatment were given to 35% patients (increased fluid intake in 19%, inhalation 18%, bed rest 8%, multiple items possible). The advice of increased fluid intake was given to 27% of patients with the prescription of acetylcysteine (which is recommended in the package insert). One quarter of smokers were advised to stop smoking.

### Sickness certification

A sickness certification was issued to 57% of the professionally active patients for a median duration of 4 days. 18% declined the proposal of a sick note.

### Patient rescheduling

43% of the patients were asked to return for a "control visit" and 21% were informed to revisit if they became worse. Patients with the diagnoses 'tonsillitis' (in 63%) and 'bronchitis' (61%) were more often asked for a "control visit" than patients with the diagnoses 'URTI/ common cold' (23%).

## Discussion

Our observational study confirmed the overuse of antibiotics and cough and cold preparations despite the lack of scientific evidence. The analysis of predictive indicators for the prescription showed that antibiotic prescribing was associated with specific patient symptoms and physical examination results whereas the prescription of cough and cold preparations was mostly performed indiscriminately. Efforts to reduce antibiotic prescribing, e.g. recommendations for self-medication, counselling on home remedies or delayed antibiotic prescribing were rare.

### Consultation rates for RTI

RTI consultation rates of up to one fifth of all patients seen a day may appear high when compared internationally [17]. However, contacting a GP with symptoms of RTI without appointment is a usual and frequent behaviour of German patients, who tend to frequently consult even for minor complaints. Both cultural factors and a fee-for-service oriented healthcare system are likely to influence consultation rates [18]: On average, German patients visit their GP two times often than e.g. people from the Netherlands or France and even 3 times often than in Sweden [17]. Consequently German GPs see many more patients per day than most of their foreign colleagues. As to our study, one-day visits proved to be sufficient to acquire a representative number of patients.

### Antibiotic prescription

In our study, the demonstrated antibiotic prescription rate was high in comparison to other European investigations and rather approaches US-American levels [2,8]. Parallel to this observation study, a documentation study was performed in Germany using questionnaires to record GPs' behaviour with patients also displaying symptoms of RTI [8]. The results of these two studies differ although the patient characteristics and diagnoses were comparable. One explanation for the lower prescription rates in the documentation study could be a selection bias because all of the GPs in our observation study were randomly recruited, whereas all of the GPs participating in the documentation study were recruited at an EBM-workshop. In addition, the completion of the documentation sheets in the sense of "scientific acceptability" is a known confounder [8,9]. However, it can be argued that the direct observation method also influenced the prescription rate ("observer effect" or "Hawthorne effect") [19]. It appears likely that the intense public discussion in the last years about the overuse of antibiotics in primary care would rather lower the rates.

In our observation, patient complaints and physical findings such as 'pathologically altered tonsils' or 'wheezing/rales' in lung auscultation have been shown to be strong predictors of GPs prescribing behaviour comparable to recent investigations [5,6]. Besides clear evidence-based medical facts, more unspecific factors such as fever or fatigue caused GPs to prescribe antibiotics, as well. Furthermore, 'yellow sputum' was positively associated with antibiotic prescription as demonstrated in other studies before [8], although this symptom has shown to be unspecific in predicting bacterial infections (in acutely ill patients) [20,21]. It appears that GPs' behaviour is influenced by empirical "internal evidence" (to detect bacterial infections), which – at least partially -, contradicts actual scientific "external evidence" [4,5,22]. The extensive impact of those predictors (explaining 70% of the variance of antibiotic prescribing) demonstrates that efforts to reduce high antibiotic prescribing rates in RTI must focus on GPs' beliefs in pathogenesis and the misattributing of clinical signs and patient symptoms to bacterial infection.

Diagnoses made by GPs were strong predictors of prescribing behaviour. Patients with the diagnosis 'URTI/ common cold' received significantly fewer antibiotics than those with 'tonsillitis', 'sinusitis' or 'bronchitis'. Despite the controversial scientific evidence for antibiotic therapy in those conditions, the majority of participating GPs seemed to be convinced that antibiotics were necessary or likely to be helpful [4,5,22]. Furthermore, patients with these diagnoses were frequently rescheduled for a "control visit" probably reflecting GPs' beliefs about "potentially dangerous diseases".

Complementary to quantitative analysis direct observation offered interesting insights in the GP-patient-consultation. Only one patient asked for an antibiotic prescription, which is in contrast to the common belief about patient expectations [3,23]. Surprisingly, the observer noticed only few efforts of GPs to reduce antibiotic prescription rates. The questionable benefit of antibiotics was discussed in very few consultations only and no doctor used delayed prescribing which has been demonstrated to be a useful tool to reduce antibiotic intake [24].

The impact of non-medical reasons of prescribing should not be disregarded [25]. Our observational study survey could not measure the impact of tacit or implicit beliefs and relational aspects on the very complex issue of motivation for prescribing. However, the importance of non-medical reasons (e.g. patients' expectations or GPs perception of those expectations) as an independent predictor of antibiotic prescribing has already been shown in several studies [3,5,7,23,26]. Additional influences of the healthcare system seem probable: fee-for-service remuneration, liberal access to GPs and specialist may result in GPs fearing to lose patients (and money) when denying a prescription.

### Prescription of cough and cold preparations

Prescription of cough and cold preparations in patients with acute RTI is not supported by scientific evidence [27-29], in particular for the expectorants acetylcysteine and ambroxole, which are the most commonly used cough and cold preparations in Germany. Although some investigations have demonstrated an effect of those substances on chronic bronchitis, evidence in acute RTI is so far absent because well-performed control trials of ambroxole and acetylcysteine are rare [30].

International comparisons of OTC-prescription rates are restricted because of different health insurance systems (and refunding policies) in different countries. Recent data from Belgium – a country with an at least partially comparable health system – showed high prescription rates for cough and cold preparations, too [31]. Interestingly, Belgium GPs preferred prescribing of cough suppressants rather than expectorants, which is the leading group in Germany (Table 2).

The fact that only 19% of the variance of cough and cold preparation prescriptions was explained by clinical signs and patient symptoms supports the assumption that non-medical reasons dominated the decision to prescribe. Frequently, the term "pseudo-placebo" is used in this context to describe the function of those preparations [32] and assumed patient expectations were mainly held responsible for the prescription [3,23].

Eighty-three percent of patients asked confirmed the use of OTC-preparations before the consultation, which were often identical with the subsequently prescribed preparations. GPs did not appreciate the opportunity to encourage this self-medication and cost reducing patient behaviour, perhaps because they were afraid that patients would not consult them with similar complaints at another time [33]. In this context, it must be noted that, in contrast to the UK, German GPs are paid only for patients actually consulting them.

## Conclusions

The method of direct observation allowed for detailed and sensitive insights in GP-patient-consultations. Nevertheless, investigators must be aware of the specific disadvantages of the method (e.g. the "observer effect").

Furthermore, we could demonstrate that patient complaints and physical examination results had a strong impact on GPs prescribing behaviour, especially on antibiotic prescription. Although this GP behaviour is not in accordance with actual scientific evidence, GPs' understanding of pathogenesis and the value of clinical signs should be strongly considered when efforts are made to reduce the overuse of antibiotics in primary care.

## List of abbreviations

ATC code = "Anatomic, Therapeutic, Chemical Classification" code

CI = Confidence interval

GP = General practitioner

OR = Odds ratio

RTI = Respiratory tract infection

URTI = Upper respiratory tract infection

## Competing interests

The author(s) declare that they have no competing interests.

## Authors' contributions

TF participated in the study design, analysis, interpretation of data and drafted the manuscript. SF participated in the study design, analysis, interpretation of data, draft of manuscript and performed the practice observations. MMK participated in the study design and draft of manuscript. EHP participated in the study design, analysis, interpretation of data and draft of the manuscript. All authors read and approved the final manuscript.

## Pre-publication history

The pre-publication history for this paper can be accessed here:


